# The Transmission of *Campylobacter* Strains in Dairy Herds in Different Housing Systems

**DOI:** 10.3390/pathogens13040317

**Published:** 2024-04-12

**Authors:** Beata Wysok, Małgorzata Rudowska, Agnieszka Wiszniewska-Łaszczych

**Affiliations:** 1 Department of Veterinary Public Health, Faculty of Veterinary Medicine, University of Warmia and Mazury in Olsztyn, Oczapowskiego 14, 10-718 Olsztyn, Poland; 2 Department of Animal Reproduction with Clinic, Faculty of Veterinary Medicine, University of Warmia and Mazury in Olsztyn, Oczapowskiego 14, 10-718 Olsztyn, Poland

**Keywords:** *Campylobacter*, dairy farms, bulk tank milk, virulence genes, AMR

## Abstract

Cattle are frequent carriers of *Campylobacter* spp.; therefore, these bacteria may be transmitted to humans through meat or milk. *Campylobacter* spp. in raw milk derives most commonly from secondary fecal contamination during the milking process; however, the udder excretion of *Campylobacter* may be a cause of milk-borne infection. Studies were carried out on a *Campylobacter*-positive farm with two different housing systems (with free-stall and tie-stall systems). The sampling process comprised several stages, including samples being taken from animals, such as from raw milk and feces, and from the environment, such as the from floor in the milking parlor and from teat cups. None of the individual raw milk samples or swabs from the floor in the parlor before the milking process were positive for *Campylobacter* spp. Simultaneously, *Campylobacter* spp. was isolated from all swabs from the floor after the milking process and in the bulk tank milk samples from the two farms. The incidence of *Campylobacter* isolated from fecal and teat swab samples ranged from 15.4% to 26.7% and from 8.9% to 25%, respectively. Altogether, 59 recovered *Campylobacter* isolates were classified, based on sequencing of the *flaA* short variable region, showing 15 different allele types, and the majority of them were distributed among one farm. Analysis of the virulence and antimicrobial properties showed that genes related to adherence, invasion and cytotoxicity were widely distributed among the *Campylobacter* recovered strains. In relation to AMR, multidrug resistance was noted in 16.1% of strains.

## 1. Introduction

In recent years, an immense increase in the number of foodborne diseases in humans caused by *Campylobacter* spp. has occurred. According to the World Health Organization (WHO), *Campylobacter* is one of four key global causes of diarrheal diseases worldwide [1]. In 2022, EU/EEA member states reported 137,107 confirmed cases of campylobacteriosis, which corresponds with an overall notification rate at the level of 43.1 cases per 100,000 population [2]. Additionally, the notification rate remained stable with no significant decrease or increase between 2018 and 2022. Campylobacteriosis is a foodborne disease that not only significantly affects human health and life but also has economic consequences for individuals, families, society and the state. This disease poses a significant burden on healthcare systems and significantly undermines the systems’ economic viability. According to the EFSA, the annual cost of campylobacteriosis associated with public healthcare and lost productivity in the EU reaches EUR 2.4 billion. *Campylobacter* species constitute the natural microflora of the digestive tract of livestock and wild animals, which is why these bacteria are widely distributed in the environment and are isolated from various sources, including water bodies, soil and food [3]. Campylobacteriosis in humans occurs mainly through the ingestion of contaminated food, and the most common transmission route is handling and eating raw or undercooked poultry meat [4]. However, cattle are also frequent carriers of *C. jejuni* and *C. coli* and other *Campylobacter* spp.; therefore, these bacteria may be transmitted to humans through meat or milk [5]. Unpasteurized bovine milk was a frequently incriminated vehicle of campylobacteriosis outbreaks reported in Finland in 2012 [6], Utah in 2014 [7] and northwest England in 2016 [8]. According to Oliver et al. [9], the presence of *Campylobacter* spp. in raw milk can be attributed mainly to secondary fecal contamination during the milking process. Poor pretreatment of the teats with disinfectant or milking clusters that come into contact with the parlor floor may result in higher levels of fecal *Campylobacter* contamination. Simultaneously, the proven cause of the contamination of milk is direct contamination in the course of mastitis; thus, udder excretion of *Campylobacter* may be a cause of milk-borne *Campylobacter* infections [10]. The infective dose of *Campylobacter* cells is very small, and it has been estimated that as few as 100 cells could cause human illness [11]. The above implies that the presence of even a very small number of *Campylobacter* cells in milk poses a potential health hazard. The first symptoms of campylobacteriosis usually occur between day 2 and 5 after infection. Diarrhea, fever, malaise and severe abdominal pain are commonly occurring clinical signs. Fatal cases are rare and usually occur in infants, the elderly or patients with impaired immunity [3]. However, complicated *Campylobacter* infections may cause extraintestinal diseases affecting the nervous, pulmonary, immune and cardiovascular systems [12]. One serious postinfectious disease that can occur after an episode of *Campylobacter* infection is Guillain–Barré syndrome (GBS). The damage to peripheral nerves observed in the course of GBS is reported to be due to cross-reactivity between antibodies produced in response to *C. jejuni* lipooligosaccharide (LOS) and human gangliosides.

The specific virulence mechanisms of *Campylobacter* infection in humans has not yet been well defined, but several factors have been implicated in the pathogenesis of *Campylobacter* infections [13]. Many authors underline that the invasiveness of *Campylobacter* strains plays a vital role in the pathogenesis of this organism; therefore, it is often used as a measure of bacterial virulence [14]. In this process, the involvement of multiple bacterial structures and mechanisms has been described, such as *Campylobacter* invasive antigen B, which participates in the translocation of *Campylobacter* into host cells, or phospholipase A, which plays an important role in host cell penetration by hydrolysis of phospholipids in the cell membrane [15,16]. However, the internalization of cells needs an initial stage, i.e., the adherence of bacteria to host cells. Bacterial adhesion depends on many factors, including bacterial motility provided by polar flagella and adhesin production, which individually or collectively can influence or mediate the bacterial adhesion to different cell structures and in different hosts [17,18]. Another major category of virulence factors are bacterial toxins. Toxins produced by *Campylobacter* are divided into enterotoxins and cytotoxins; however, only cytolethal distending toxin (CDT), causing cell cycle arrest, has been well characterized at the molecular level.

Taking into consideration public health, it is important to not only determine the possible sources of zoonotic agents or their virulence properties but also to monitor for the occurrence of antimicrobial-resistant strains. Combined resistance to critically important antimicrobials is a significant public health issue, as multidrug resistance (MDR) constitutes a major obstacle to effective therapeutic agents.

The aim of the current study was to indicate the prevalence ratio of *Campylobacter* spp. in feces of dairy cows and to identify possible transmission routes of these bacteria to raw milk, the distribution of antimicrobial patterns and virulence-associated genes, as well as the phylogenetic diversity of the obtained isolates.

## 2. Materials and Methods

**Figure 1:**
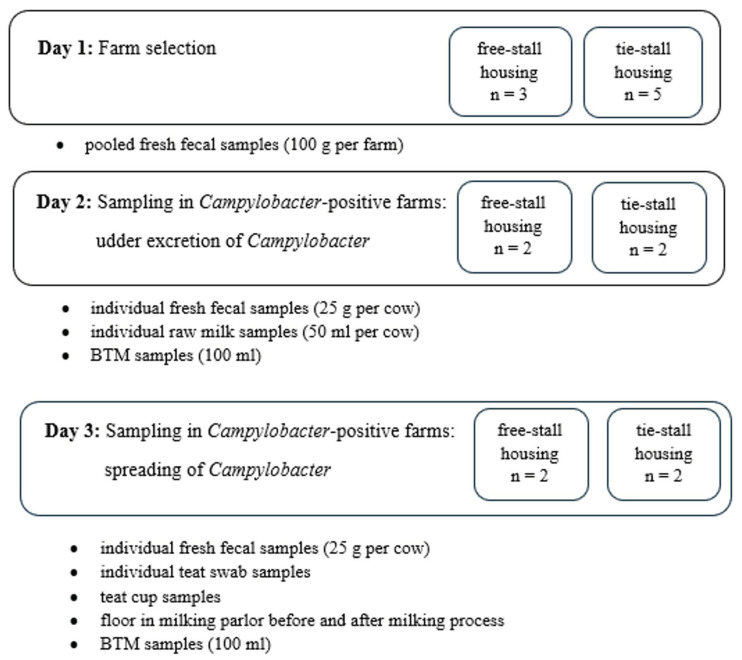
Sampling scheme.

The sampling process comprised several stages, and it is presented schematically in Figure 1.

**Farm selection.** The main aim of this study was to assess the risk associated with contamination of raw milk at *Campylobacter*-positive dairy farms with different housing systems. Cow feces sampled from eight dairy farms was tested for the presence of *Campylobacter* spp. Three farms had a free-stall housing system (the animals were confined together on deep litter), and five farms had a tie-stall housing system (the cows were tied in their own stall for the duration of their lactation and could go outside or out to pasture during the dry period, and they were kept on rubber mats without straw bedding). All the farms tested were situated in the Warmia and Mazury and Mazowsze regions (Poland). The size of herds ranged from 15 to 120 cows. Pooled fresh fecal samples were collected from fresh manure from 5 spots on the floor. Routine *Campylobacter* culturing was performed with 100 g of pooled fecal samples according to the protocol described below. All the *Campylobacter*-positive flocks, selected at this stage, were included in the further study. Simultaneously, bulk tank milk (BTM) samples were obtained to determine its significance in terms of human campylobacteriosis. The procedure for the sampling of BTM is described below.

**Characteristics of *Campylobacter*-positive farms.** The characterization of the tested *Campylobacter*-positive dairy farms and the characterization of the teat disinfectant products are presented in Table 1. On every tested farm, the process of obtaining milk was based on a milking machine. On all the farms before milking, the udder and the teats were cleaned by a single-use towel for each individual cow, and then the health control of the lactating dairy cows was performed by visual examination of the foremilk from each teat stripped out into a strip cup. Teat pre-dipping was performed on none of the tested farms, and on all farms, post-milking teat disinfection was carried out. The total bacterial count (TBC) and somatic cell count in the milk (SCC) of the bulk tank milk samples collected were determined using a BactoCount IBCm apparatus (Bentley Instrument, Minnesota, USA). In addition, cows with symptoms of clinical mastitis treated through intramammary infusion of antibiotic ointments were excluded from the study.

**Sampling in *Campylobacter*-positive farms.** As the study was undertaken to establish the possible routes of milk contamination with *Campylobacter* spp., the sampling was performed in the two following days. On the first day, the possibility of raw milk contamination due to subclinical mastitis caused by *Campylobacter* spp. was examined. Individual fresh fecal samples and raw milk samples as well as bulk tank milk (BTM) samples were taken. A total of 25 g of feces samples was obtained from the recta of the cows with a single-use disposable obstetric glove lubricated with sterile water. The feces samples were placed into sterile plastic cups. After udder disinfection with sulfonic acid and after discarding the first squirt of milk, raw milk samples were obtained from all quarters and pooled in 50 mL sterile Falcon tubes. On each farm, one BTM sample in a volume of 100 mL was taken after morning milking from a tap connected to the cooling tank once the milk was cooled to a temperature of +4 °C.

On the second day, the contamination of raw milk during the milking process was examined. Before the animals were moved to the milking parlor for morning milking, fecal samples and teat swabs from each individual cow, both lactating and dried ones, were taken. The feces samples were obtained as described above. Teat swab samples were taken with sterile cotton pads (one per teat) immersed in 0.9% NaCl. The swabs were taken from the teat end skin whilst avoiding contact with udder hair before mechanical cleaning of the teats by a single-use towel, and the samples were pooled in aseptic stomacher bags with 25 mL of NaCl solution. Simultaneously, environmental swabs from the teat cups and the floor in the milking parlor as well as the bulk tank milk (BTM) were taken. The teat cups (composed of a rigid outer shell and a soft inner liner that was in contact with teat) were swabbed from the external and internal surfaces before milking with sterile cotton swabs immersed in 0.9% NaCl, and the swabs were subsequently immersed in 10 mL of NaCl solution in 15 mL Falcon tubes (Sarstedt). Floor swabs from four different places in the milking parlor were collected with a sterile sponge immersed in 0.9% NaCl. Two separate swabs were taken, before and immediately after the milking process. The sponges after swabbing were placed in aseptic stomacher bags with 25 mL of NaCl solution. The BTM samples were obtained as described above.

**Processing of samples.** All samples were kept at +4 °C, transported to the laboratory and analyzed within 8 h of collection. The feces samples, the pooled teat swab samples, the pooled teat cups swab samples and the floor swab samples were transferred to ninefold volumes of Bolton broth (Oxoid, Basingstoke, UK). The enrichment cultures were incubated in a microaerophile atmosphere (85% N_2_, 10% CO_2_, 5% O_2_) at 37 °C for 4 h and next at 42 °C for 44 ± 4 h. The cultures obtained in the broth medium were transferred using a sterile loop to the surface of two parallel selective agar media: mCCDA (modified *Campylobacter* Blood-Free Selective Agar Base, Oxoid) and Karmali (Oxoid). The plates were incubated at 41.5 °C in a microaerophile atmosphere. After 44 ± 4 h of incubation, the plates were checked for the presence of colonies suspected of belonging to the genus *Campylobacter*. From each sample, three characteristic grayish, flat, moist colonies with the tendency for overflowing growth were analyzed under a contrast-phase microscope (1500× magnification) and were chosen for further testing. If two isolates of the same origin belonged to the same *flaA*-SVR allele and showed the same antimicrobial resistance pattern, they were considered as the same strain, and only one isolate was chosen for further analysis.

The isolation procedure for *Campylobacter* spp. from the individual milk samples and bulk tank milk was carried out regarding the method described by [19]. The pH of the milk was determined and established at the final level of 7.6 by the addition of 1–2 M NaOH. A total of 50 mL of the raw milk samples was centrifuged at 20,000× *g* for 40 min. The supernatant was discarded, and the pellet was dissolved in 10 mL of Bolton broth, and then the pellet was transferred to 90 mL of Bolton broth. The suspension was incubated under microaerophilic conditions in accordance with the procedure described above.

Subsequently, the isolates obtained were subcultured only once in order to minimize the changes resulting from several passages and were stored at −80 °C in defibrinated horse blood (Oxoid, Basingstoke, UK) with added glycerol (80:20 *v*/*v*).

**Species identification.** Species identification of the isolates was carried out based on the primers and amplification procedure listed in a previous study [20]. The PCR product was run on a 2% agarose gel stained with ethidium bromide at a concentration of 5 μg/mL. The size of the amplification product was determined using a 100 bp molecular weight marker.

**Detection of virulence-associated genes.** The identification of virulence-associated genes responsible for adhesion and colonization (*flaA*, *cadF* and *racR*), responsible for invasion (*virB11*, *iam*, *ciaB* and *pldA*), responsible for the production of cytotoxins (*cdtA*, *cdtB* and *cdtC*) and Guillain–Barre-syndrome-associated genes (*cgtB* and *wlaN*) was performed based on the primers and procedure listed in a previous study [20].

**Sequencing of *flaA*-SVR.** The DNA of all the isolates obtained in this study was subjected to *flaA* short variable region (SVR) and sequencing using the primers FLA242FU and FLA625RU [21]. For PCR, the conditions were as described above, with the annealing temperature specific for a given primer pair set at 53 °C. The PCR products were visualized via gel electrophoresis, purified with a Clean-Up Kit (A&A Biotechnology, Gdańsk, Poland) and sequenced by Sanger sequencing (Genomed, Warszawa, Poland). The forward and reverse sequences were assembled using the Contig Express module in Vector NTI Express (Thermo Fisher Scientific, Waltham, MA, USA) and trimmed to a 321 bp length covering the *flaA*-SVR. The sequences were assigned *flaA*-SVR allele numbers according to the PubMLST database (http://pubmlst.org/campylobacter, accessed on 15 June 2023), and a cluster analysis was then performed using the default parameters in MEGA X v. 10.1 (http://www.megasoftware.net, accessed on 20 January 2024). The maximum likelihood tree based on the *flaA*-SVR sequences was visualized in iTOL v4 (https://itol.embl.de, accessed on 20 January 2024). The obtained sequences were submitted to the GenBank database and received the following accession numbers: PRJNA1085630.

**Antimicrobial resistance (AMR).** Antimicrobial resistance was examined by the minimal inhibitory concentration (MIC) method using the agar dilution method. Inocula were prepared in Mueller–Hinton broth (Biomaxima, Lublin, Poland) at a density adjusted to a 0.5 McFarland turbidity standard and diluted 1:10 to achieve a final concentration of 10^4^ cfu/mL. Using a Steers multipoint replicator, the inocula were transferred onto previously prepared Mueller–Hinton agar with serial twofold dilutions of each antimicrobial agent from 0.015 to 64 mg/L (for erythromycin and ciprofloxacin) and from 0.03 to 128 mg/L (for ampicillin, tetracycline and gentamicin). The plates were incubated in a microaerobic atmosphere for 24 h. The MICs were determined to be the lowest concentration of the antibacterial chemical that showed no visible growth of the target organism. The control used for AST was the standard bacterium *C. jejuni* ATCC 33560. The MICs of inhibited growth for erythromycin, ciprofloxacin and tetracycline were determined according to the EUCAST breakpoints for *Campylobacter*. For the remaining tested antimicrobials not specified for *Campylobacter* by EUCAST, we used the breakpoints for Enterobacteriaceae.

**Statistical analysis.** Statistical differences in the presence of *Campylobacter* isolates in the samples collected from cattle and in the presence of virulence genes were determined using a 2 × 2 contingency table and Fisher’s exact test (Statistica, Kraków, Poland). The level of significance was set at *p* < 0.05.

## 3. Results

**Prevalence of *Campylobacter* strains in the dairy farm.** Of the eight tested dairy farms, four (50%) were positive for *Campylobacter*, including two out of three (66.7%) farms with a free-stall housing system and two out of five (40%) farms with a tie-stall housing system. 

**Distribution of *Campylobacter* strains in animal and environmental samples obtained in *Campylobacter*-positive dairy farms.** Among the tested farms, none of the individual raw milk samples were positive for the presence of *Campylobacter* spp. (Table 2). At the same time, *Campylobacter* spp. was recovered from the BTM samples obtained from the two farms tested, with no difference in testing on two separate days. *Campylobacter* spp. was detected in similar levels on each farm tested in the rectal swab samples (*p* > 0.05), and the values ranged from 15.4% in Farm B to 26.7% in Farm D. Simultaneously, the isolation rate of *Campylobacter* spp. from the teat swab samples differed significantly between the farms, and the noted values ranged from 8.9% in the swabs in Farm B to 25% in the swabs in Farm A (*p* < 0.05). In all the farms, the teat cup swabs and floor swabs before the milking process were *Campylobacter*-negative; however, the prevalence of *Campylobacter* spp. was confirmed in the floor swabs samples of all the tested farms after the milking process.

**Figure 2:**
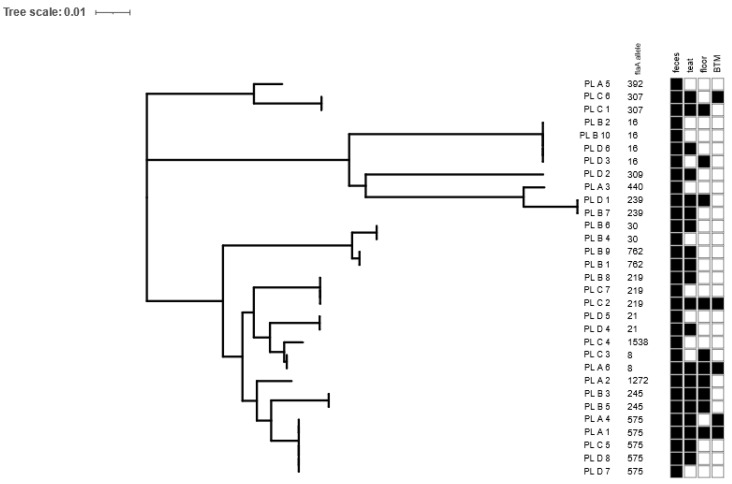
Maximum likelihood tree of *Campylobacter* flaA-SVR allele sequences among isolates originating from dairy farms. For each isolate, the following characteristics are shown: strain ID (according to the following pattern: country of isolation, code of farm, individual number of tested sample), *flaA* allele number and sample type (feces, teat, floor and bulk tank milk (BTM)). The distribution of *Campylobacter*-positive samples in relation to sample type is indicated by black (present) and white (absent) squares. The figure was visualized in the interactive Tree of life (iTol).

**Genetic diversity.** The identification of species based on PCR showed that all the obtained isolates belonged to *Campylobacter jejuni*. The 59 recovered *Campylobacter* isolates were classified to 31 different strains (based on the *flaA*-SVR allele and antimicrobial resistance pattern), which were assigned to 15 *flaA* allele types. In the majority of the positive fecal samples and teat swabs, only one strain was isolated, apart from two swabs from teats (one on farm A and one on farm D) and one fecal sample (on farm A), which were contaminated with two distinguishable *Campylobacter* strains. Simultaneously, among the positive floor swabs and bulk tank milk samples, two and three strains, respectively, were found. Only 5 out of the 15 *flaA* allele types were distributed among the different farms, and therein, allele 575, covering five isolates, was present in three out of the four tested farms (farms A, C and D). The remaining alleles were found in only one flock, and 50% of them occurred only once (Figure 2). On each of the tested farms, in the floor samples after the milking process and in the bulk tank milk, recovered *Campylobacter* isolates were previously found in the rectal swabs and/or teat swabs. Altogether, over 60% of the dairy cows shedding *Campylobacter* via feces showed microbial contamination of the teat skin. Only a single negative-culture cow showed the prevalence of *Campylobacter* on the cow’s teats.

**Figure 3:**
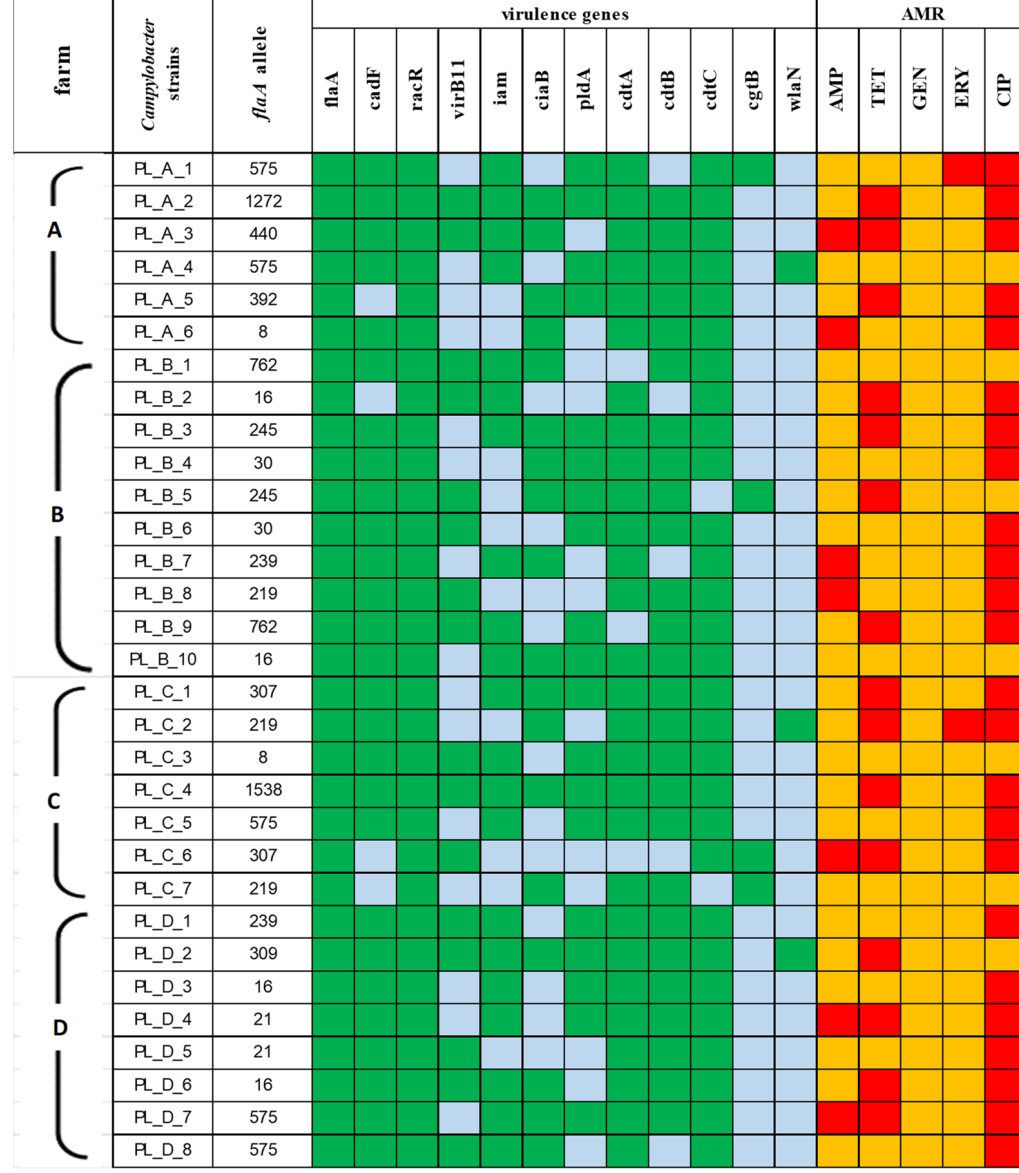
Heat map with the prevalence of virulence genes and the antimicrobial resistance (AMR) frequency among *Campylobacter* strains by the four farms (A, B, C, D) of origin. In relation to virulence genes, present genes are shown in green and absent genes in blue. In relation to AMR, the strains resistant to antimicrobials tested are given in red and susceptible strains are given in yellow.

**Distribution of pathogenic genes.** Regardless of the source, no significant differences regarding virulence patterns were observed among the recovered *Campylobacter* strains. The majority of the isolates harbored virulence genes associated with adherence and cytotoxicity, and the patterns *flaA*_*cadF*_*racR* and *cdtA*_*cdtB*_*cdtC* were present in 87.1% (27/31) and 70.9% (22/31) of the strains, respectively. The prevalence rates of invasion-related genes showed significant divergence, as 12 different patterns were noted. The gene frequency was in the range from 51.6% for *virB11* to 67.7% for *iam*. The pattern covering all the tested genes *virB11_iam_ciaB_pldA* was noted in 9.7% (3/31) of the isolates obtained. Only a few isolates possessed *cgtB* (4/31, 12.9%) and *wlaN* (3/31, 9.7%) genes related to GBS (Figure 3).

**Antimicrobial resistance.** The highest resistance rate was observed to ciprofloxacin (77.4%), while none of the *Campylobacter* strains were resistant to gentamicin (Table 3). The prevailing resistance pattern was TET_CIP (25.8%). Only 4 out of the 31 isolates recovered were susceptible to all the tested antimicrobial agents. Multidrug resistance, determined as resistance to at least three different classes of antimicrobials, was detected in five isolates, i.e., to AMP_TET_CIP in four isolates and to TET_ERY_CIP in one isolate (Figure 3).

## 4. Discussion

As milk is a product of exceptional nutritional value, it is an important element of consumers’ diets. However, despite its unique composition and properties, milk is an excellent medium for bacterial growth and source of bacterial infection [22]. According to Ouamba et al. [23], the microbiological status of raw milk is affected by several factors, including animal health, the farm environment and management practices. Mainly, the presence of *Campylobacter* in raw milk is primarily due to fecal contamination of teats and udders. In our study, the contamination of bulk tank milk was significantly correlated with colonization of the intestinal tract, as culture-positive BTM samples were observed only in the dairy farms with confirmed shedding of *Campylobacter* in the feces. Overall, the prevalence of *Campylobacter* is considered to be common in cattle herds, and 50% of the dairy herds tested in our study showed at least one cow shedding *Campylobacter*. This result is similar to that noted by Hoque et al. [24] in Bangladesh (53.3%); however, the farm-level occurrence rates of these bacteria reported in different geographical locations were variable, ranging from 4% in Portugal [25] to 35.7% in Italy [26] and to 60.0% in South Korea [27]. Interestingly, Klein et al. [28] described a few factors that can increase the possibility of the appearance of *Campylobacter* spp. in cattle farms. One is, among others, the presence of poultry on the farm, as poultry are known to be the most important reservoir of *Campylobacter*. Simultaneously, no association was shown between *Campylobacter* in cattle and the presence of other animals such as sheep, goats, pigs, equines and pets on farm. However, findings suggest that wild birds may play a role in sustaining the epidemiology of *Campylobacter* spp. on farms [29]. It is worth remembering that wild birds feeding on the remains of food of animal, plant and mixed origin most often stay close to farm animals and human habitats, which is why they are more exposed to *Campylobacter* spp. infection than those feeding further away or hunting in the air. At the farm level, both clinically and asymptomatically, infected animals harboring *Campylobacter* may shed bacteria, thus increasing the risk of infection of other animals or humans through contamination of the environment [27,30]. In the current study, the rates of shedding of *Campylobacter* among the dairy cows within the farms with tie-stall and free-stall housing systems ranged from 15.4% to 26.7%, respectively, depending on the housing system. More convenient conditions for cross-contamination when animals are kept on the deep floor of a free-stall housing system is a possible explanation for these findings. Also, the studies performed by Idland et al. [31] underlined that samples collected from loose housing systems had a significantly higher content of *L. monocytogenes* and *Campylobacter* spp. than samples collected from tie-stalled herds, simultaneously suggesting that the type of housing system may influence the food safety of raw milk, as one infected calf can contaminate the environment, which leads to a quick transmission of campylobacters among calves of the same group [32]. On the other hand, van Aken et al. [33] suggest that free-stall housing, in combination with increased lying comfort, can have a positive effect on udder health and animal welfare, with lower incidences of clinical mastitis. It should be noted that the colonization of the gastrointestinal tract by *Campylobacter* spp. results in its shedding and contributes to the contamination of the outside of the udder and teats. In our study, out of the 33 dairy cattle that were positive for *Campylobacter* spp. in their fecal samples, the contamination of teat skin by these bacteria was observed in 64.5% of the tested animals. Bearing in mind the milking process, the need to maintain strict hygienic standards to prevent the contamination of milk with bacteria from the surface of the teats should be emphasized. At the same time, it is worth adding that mechanical cleaning of teats by wet wipes before the milking process without any disinfection of teats during the pre-dipping process poses a risk for further contamination of raw milk. It has been underlined that udder hygiene is one of the most important variables resulting in high microbiological milk quality. Poor hygiene is reflected by the high proportion of samples contaminated with Staphylococcus aureus and E. coli noticed in a study performed by Knight-Jones et al. [34], suggesting poor handling and fecal contamination. Simultaneously, in the current study, the udder excretion of *Campylobacter* spp. in the course of asymptomatic mastitis has not been shown. However, some authors have noticed that mastitis occurs when the teats of cows are exposed to pathogens that penetrate the teat duct and establish an infection in one or more quarters within the udder.

Our results showed a high diversity in the *Campylobacter* isolates, indicating the occurrence of unique *flaA* sequence types among the tested dairy farms. Out of the 15 *flaA*-SVR sequences covering 31 of the *C. jejuni* strains, only five alleles (33.3%) were present on the different farms. The prevailing was allele type 572, covering 5 of the 31 isolates derived from three out of the four tested farms. Based on the data derived from the pubMLST database (https://pubmlst.org/, accessed on 11 February 2024), we noticed that this sequence is specific to human, swine and cattle sources. Simultaneously, at the farm level, a wide dissemination of isolates was observed, as the same isolates were found in the fecal samples, skin teats, floor samples after milking and BTM samples. In addition, at the farm with the tie-less system, contamination of teat skin by different isolates was observed, which proves that *C. jejuni* easily spreads in the environment. Also, the samples from the floor after the milking process and the BTM samples were contaminated with two or three different isolates belonging to different *flaA*-SVR alleles and representing different antimicrobial profiles. Therefore, Bianchini et al. [10] underlined that if *Campylobacter* is shed in the feces of dairy cattle, it could be easily transmitted to humans through dairy products such as unpasteurized milk.

Our study, along with the research of other authors, confirmed the virulent properties of *Campylobacter* isolated from cattle, as high prevalence rates of virulence genes that are potentially responsible for adhesion, invasion and cytotoxic activity were demonstrated. The vast majority of the isolates (87.1%) carried three tested genes, *flaA_cadF_racR*, which are associated with adhesion to and colonization of intestinal epithelial cells. Referring to virulence factors associated with invasion, only a few strains, 9.7%, possessed the profile *virB11_iam_ciaB_pldA*, covering all the tested genes. Generally, it has been described that the majority of factors that are important for microbial pathogenesis are widely distributed among *Campylobacter* isolates originating from cattle [4,35]. Furthermore, according to Lopes et al. [36], the rate of bacterial invasion does not seem to be solely responsible for the cytopathic effect associated with *Campylobacter* infection, and toxins are likely to be associated with the disease course. Cytolethal distending toxin (CDT), composed of three subunits, CdtA, CdtB, and CdtC, is the best characterized among the toxins produced by *Campylobacter* strains [37]. We found an extremely high prevalence rate of three adjacent genes encoding CDT among the *Campylobacter* strains in the current study, as they were found in 70.9% of the isolates and, simultaneously, in three out of the five found in the bulk tank milk. In vitro and in vivo studies have clearly shown that this toxin has a strong effect on cellular physiology, a.o., inflammation, immune response modulation and tissue damage [38]. Many authors suggest that the course of campylobacteriosis is unpredictable; however, the ability to produce cytotoxins is probably involved in the severity of the course of diseases caused by *Campylobacter*. Taking into consideration the virulence properties of *Campylobacter* strains, it is crucial to estimate the prevalence of virulence factors associated with post-campylobacteriosis infection, such as Guillain–Barré syndrome (GBS). Although uncommon, GBS is an acute polyradiculo-neuropathy that typically develops after a previous gastrointestinal or respiratory infection, and *Campylobacter* is firmly established as a causative agent of this syndrome [39]. The potential GBS markers, both involved in LOS sialylation and crucial for the induction of anti-ganglioside antibodies, are the *cgtB* and *wlaN* genes [40]. Here, we revealed the presence of these factors in 12.9% and 9.7% of the isolates recovered, including each time in two *Campylobacter* isolates originating from milk. According to Muller et al. [41], *wlaN* can probably overcome the lack of *cgtB* in *cgtB*^−^ isolates. In our study, altogether, seven isolates were recognized as positive for the presence of genes associated with GBS, and *wlaN* and *cgtB* genes were not found in the same isolates, which indicates the perception of raw milk as an important source of pathogenic *Campylobacter* strains able to cause extra intestinal manifestations.

Recently, an alarming trend of *Campylobacter’s* resistance profile has been observed. Our study confirmed that the *Campylobacter* isolates originating from cattle were mainly resistant to quinolones and tetracyclines, as 77.4% and 48.4% were resistant to ciprofloxacin and tetracycline, respectively, and simultaneously, 25.8% of the isolates derived in the dairy farm exhibited the resistance pattern CIP_TET. According to the European Union Summary Report on Antimicrobial Resistance in zoonotic and indicator bacteria from humans, animals and food in 2020–2021 [42], this resistance profile has been described as the prevailing one in *Campylobacter* from different sources and different geographical regions. At the same time, combined resistance to both ciprofloxacin and erythromycin is considered to be critically important for the treatment of campylobacteriosis. Generally, this resistance profile is noted to be rare or low in *Campylobacter* isolates from humans, poultry, pigs and calves. The current study reported that only 6.4% (Nn = 2) of the isolates were resistant to both antimicrobials. Furthermore, these isolates, belonging to *flaA* alleles 575 and 219, were found in different sources, including BTM samples, which indicates that *Campylobacter* strains derived from dairy cattle pose a potential risk to human health, as those are the antibiotics of choice for the treatment of human cases [43]. Interestingly, despite the fact that aminoglycosides are important veterinary antimicrobials and are used in all major food-producing animals to treat infections, gentamicin resistance is considered as a novel phenomenon in *Campylobacter* isolates [44]. These findings are in accordance with our study, since none of the derived isolates were resistant to this antimicrobial agent.

The importance of raw milk as a source of human *Campylobacter* enteritis was confirmed by the European Union summary report on food-borne disease outbreaks (http://dx.doi.org/10.2903/j.efsa.2013.3129) [45]. Milk and dairy products are important staples of a healthy diet; however, if pathogenic microorganisms are not removed by pasteurization, consumption of these products can represent a serious health risk [46]. Colonization of the gastrointestinal tract of cattle by *Campylobacter* spp. is of vital importance both because of the possibility of contamination of carcasses in slaughterhouses as well as of milk during milking on farms. As the pathogenicity of *Campylobacter* spp. depends mainly on the existence of virulence genes and the antimicrobial resistance mechanisms they possess, the study conducted indicates that raw milk and dairy products made from heat-untreated milk may be the cause of gastroenteritis in humans.

## Figures and Tables

**Table 1:** Characteristics of dairy farms tested.

Farm	Housing System	No. of Cows			Post-Dipping	Health Status		
		Total	Dried	With Mastitis	Type of Active Substance	Milk Production (L)	TBC *	SCC **
**A**	free-stall	20	1	-	chlorhexidine	7500	4.70 × 10^4^	110,000
**B**	tie-stall	80	9	2	iodine	9500	6.71 × 10^4^	123,000
**C**	tie-stall	45	5	1	iodine	9000	3.38 × 10^4^	99,000
**D**	free-stall	30	3	-	chlorhexidine	8000	4.33 × 10^4^	113,000

* TBC (total bacterial count per mL of milk). ** SCC (somatic cell count per mL of milk).

**Table 2:** Prevalence of *Campylobacter* spp. in dairy farm samples. The numbers given are positive sample/total sample (%). The letters given in superscript denote significant differences between farms.

Farm	Rectal Swab	Teat Swab	Teat Cup Swab	Floor Swab		BTM	Individual Milk
				Before Milking	Post Milking		
**A**	5/20 (25%)	5/20 ^A^ (25%)	0/1	0/1	1/1	1/1	0/1
**B**	12/78 (15.4%)	7/78 ^B^ (8.9%)	0/1	0/1	1/1	0/1	0/1
**C**	8/44 (18.2%)	4/44 ^B^ (9.1%)	0/1	0/1	1/1	1/1	0/1
**D**	8/30 (26.7%)	4/30 ^B^ (13.3%)	0/1	0/1	1/1	0/1	0/1

**Table 3:** Distribution of MICs of *Campylobacter* strains (N = 31) obtained in dairy farms.

Antimicrobial Agent	No. of Isolates at Each Concentration (mg/L)														Resistance (%)
	0.016	0.03	0.06	0.12	0.25	0.5	1	2	4	8	16	32	64	128	
**AMP**						2	5	2	4	5	3	3	5	2	22.6
**TET**			2	3	3	4	4	2	1	2	3	3	2	2	48.4
**GEN**		1	4	6	8	7	5								0
**ERY**		1	1	4	3	2	9	2	7	2					6.4
**CIP**				1	2	4	3	4	2	4	2	4	3	2	77.4

The shaded areas indicate the susceptibility range of each antibiotic tested.

## Data Availability

The data presented in this study are available on request from the corresponding author. The data are not publicly available as they are still being used for other research works.
